# Supplementary figures and images for: Compound-Specific 14N/15N Analysis of Amino Acid Trimethylsilylated Derivatives from Plant Seed Proteins
Source: Int J Mol Sci. 2022 Apr 28;23(9):4893. doi: 10.3390/ijms23094893 (PMC9105707; doi:10.3390/ijms23094893)

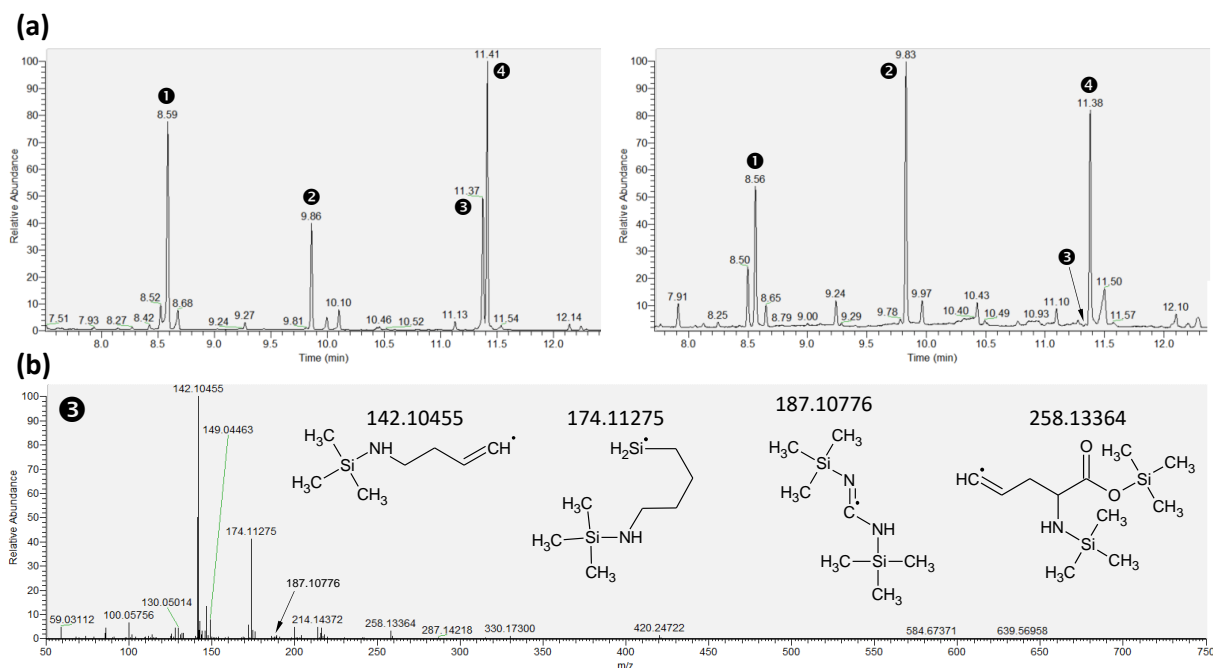

Supplement: Supplementary file 1 [file ijms-23-04893-s001.zip › ijms-1676118-supplementary.pdf]
